# Copper (0) Mediated Single Electron Transfer-Living Radical Polymerization of Methyl Methacrylate: Functionalized Graphene as a Convenient Tool for Radical Initiator

**DOI:** 10.3390/polym12040874

**Published:** 2020-04-10

**Authors:** Adhigan Murali, Srinivasan Sampath, Boopathi Appukutti Achuthan, Mohan Sakar, Suryanarayanan Chandrasekaran, N. Suthanthira Vanitha, R. Joseph Bensingh, M. Abdul Kader, Sellamuthu N. Jaisankar

**Affiliations:** 1 School for Advanced Research in Polymers (SARP)-Advanced Research School for Technology and Product Simulation (ARSTPS), Central Institute of Plastics Engineering & Technology (CIPET), Ministry of Chemicals & Fertilizers, Govt. of India, Chennai 600032, India; josephbensingh@gmail.com (R.J.B.); kader36@yahoo.com (M.A.K.); 2 Department of Materials Science, School of Technology, Central University of Tamil Nadu, Thiruvarur 610101, India; sampathsrinivasan@yahoo.com; 3 Polymer Science and Technology Division, Council of Scientific and Industrial Research (CSIR)-Central Leather Research Institute (CLRI), Adyar, Chennai 600020, India; aaboopathichem@gmail.com (B.A.A.); snjaio@yahoo.com (S.N.J.); 4 Centre for Nano and Material Sciences, Jain University, Bangalore 562112, Karnataka, India; 5 Faculty of Pharmacy and Pharmaceutical Sciences, University of Alberta, Edmonton, AB T6G 2E1, Canada; jobforsurya@gmail.com; 6 Department of Electrical & Electronics Engineering, Muthayammal Engineering College (Autonomous), Namakkal 637408, Tamilnadu, India; varmans03@gmail.com

**Keywords:** graphene, single electron transfer, radical initiator, poly(methyl methacrylate)

## Abstract

Polymer nanocomposites have been synthesized by the covalent addition of bromide-functionalized graphene (Graphene-Br) through the single electron transfer-living radical polymerization technique (SET-LRP). Graphite functionalized with bromide for the first time via an efficient route using mild reagents has been designed to develop a graphene based radical initiator. The efficiency of sacrificial initiator (ethyl α-bromoisobutyrate) has also been compared with a graphene based initiator towards monitoring their Cu(0) mediated controlled molecular weight and morphological structures through mass spectroscopy (MOLDI-TOF) and field emission scanning electron microscopy (FE-SEM) analysis, respectively. The enhancement in thermal stability is observed for graphene-grafted-poly(methyl methacrylate) (G-*g*-PMMA) at 392 °C, which may be due to the influence ofthe covalent addition of graphene, whereas the sacrificial initiator used to synthesize G-*graft*-PMMA (S) has low thermal stability as analyzed by TGA. A significant difference is noticed on their glass transition and melting temperatures by DSC. The controlled formation and structural features of the polymer-functionalized-graphene is characterized by Raman, FT-IR, UV-Vis spectroscopy, NMR, and zeta potential measurements. The wettability measurements of the novel G-*graft*-PMMA on leather surface were found to be better in hydrophobic nature with a water contact angle of 109 ± 1°.

## 1. Introduction

Graphene is a versatile class of two-dimensional carbon based macromolecule with single atom thickness [1]. Graphene and their related carbon-based materials have potential application in energy storage [2], biomedical devices [3], sensors [4], nanoelectronics [5], catalysis [6], and drug delivery [7]. Due to their high surface area to volume ratio, grpahene is being used to prepare high strength polymer nanocomposites with extraordinary physical, chemical, and mechanical properties [8]. The functionalization of graphene has received much attention in last two decades towards developing good interfacial interaction to incorporate other molecules including organic and inorganic hybrid polymers [9]. Graphene and nanotubes can act as fillers in polymer matrix in order to induce the elasticity, strength, modulus, remarkable electrical and better thermal conductivity [10]. The modification of graphene can be two categories: one is physical adsorption, i.e., the non-covalent approach or wrapping of polymer, while another is the covalent approach or polymer grafting, which improves their interfacial interaction and dispersion properties [11]. Graphene on chemical exfoliation can produce graphene oxide (GO) with several hydrophilic groups such as -COOH and -OH. This can promote the intercalation of water molecules into the graphene gallery and can be easily detached from other molecules upon ultrasonication. Thereby, the production of highly dispersible GO nanosheets in polar and non-polar solvent medium can become largely feasible [12,13,14,15]. Besides, the enhanced solubility of graphene can help exploring the solution phase-based properties as well. Hence, the functionalization of graphene with appropriate functional groups is essential for the synthesis of high-performance polymer nanocomposite and it also tends to enhance their physico-chemical properties as well [16,17,18]. The single electron transfer-living radical polymerization (SET-LRP) technique is one of the promising methods to produce the well-controlled molecular weight polymers. The SET-LRP method is less liable to side chain reactions and termination. In addition, some of the functions of this method can occur by outer sphere electron transfer, which is accepted to occur via inner sphere electron transfer mechanism, where the activation of R-X by Cu(I) and ensuing generation of Cu(II) is adopted through a halogen bridged transition state [19]. Recently, Nguyen and Percec [20] established an innovative and simple methodology involving the activation of Cu(0) wire for the spectacular acceleration of SET-LRP of methyl acrylate monomer towards getting the excellent control over molecular weight distribution and high end chain functionality. Moreover, the disproportionation versus non-disproportionation that happened during activated and deactivated Cu(0) in SET-LRP of methacrylate have also been reported recently [21]. The SET-LRP of acrylates monomer in different organic solvents, which can be mediated various degrees of disproportionation of Cu(I)Br, have also been reported. Percec et al. [22] has also demonstrated a novel and smart methodology involving a binary mixture of solvents in SET-LRP of acrylates. Matyjaszewski et al., reported that variety of technique which include SET-LRP and supplemental activator and reducing agent atom transfer radical polymerization (SARA ATRP) are demonstrated with different monomers to get high molecular weight polymers [23,24]. Recently, our group also demonstrated the SWCNT-*graft*-PMMA used for the polymer stabilized liquid crystal devices (PSLC) [25,26]. Based on these insights, we have developed a polymer nanocomposite by the covalent addition of bromide-functionalized graphene through SET-LRP technique, where we have used the graphene as an active radical initiator that derived from the bromide functionalized graphite via an efficient route using mild reagents for the first time. In addition to this, a bromide functionalized graphene based radical initiator has been synthesized in order to investigate the leeway of well controlled grafting of PMMA on graphene surface. The effect of graphene-initiator on the controlled polymerization was compared with the sacrificial initiator using ethyl bromoisobutyrate. Furthermore, there is a demand for highly hydrophobic materials for the fundamental technologies in antistatic phenomena, self-cleaning and anti-pollution mechanisms. We demonstrated the graphene-*g*-PMMA with the enhanced dewetting properties on the leather surface. Notably, graphene substrates do not affect the wettability due to the large intermellar coupling between the graphene-based composites and the underlying leather substrates [27,28,29]. Hence, we have focused on the hydrophobic coating of various leather accessories.

## 2. Experimental Section

### 2.1. Material

Methyl methacrylate (MMA) monomer was purchased from Sigma-Aldrich (Bangalore, Karnataka, India) and the monomer was purified by passing it through an Al_2_O_3_ column (elimination of inhibitor) before use. Graphite powder (conducting grade, 325 mess, metal basis and 99.99%) was obtained from Alfa Aesar (Chennai, Tamilnadu, India). Bromoisobutyryl, bromide, *N*,*N*,*N^I^*,*N^II^*,*N^III^*-Pentamethyl diethylene triamine (PMDETA), Ethyl bromoisobutyrate (sacrificial initiator) and Copper (0) powder were procured from Sigma-Aldrich and used as received. Organic solvents (triethylamine (Et_3_N), tetrahydrofuran (THF), Dimethyl sulfoxide (DMSO), dimethyl formamide (DMF), and hexane were dried and doubled distilled as per the typical protocols before use.

### 2.2. Methods

Raman spectra were recorded on a confocal Raman spectrometer (Nano photon Corporation, Suita, Osaka, Japan, excited at 532 nm by using laser of Ne). The NMR was recorded on a Jeol 500 MHz spectrometer (Peabody, MA, USA); all the spectra in were recorded in CDCl_3_ and DMSO d_6_ solution at room temperature. Matrix assisted laser desorption/ionization (MALDI), model: MICROFLEX LRF Bruker (Preston, Victoria, Australia) was used to determine the molar mass distribution of the synthesized polymer. The field emission scanning electron microscopy (FE-SEM) from FEI Quanta 200(Hillsboro, OR, USA), operated at 30 kV was used to obtain the images. The size distribution and zeta potential analyses were performed on a Malvern, model no: Nano-ZS (Cambridge, UK). Thermal analysis was performed using DSC Q-200 and TGA Q-50, TA instruments, WATERS, Vienna, Austria.

### 2.3. Synthesis of [NO_2_]_n_-Graphene from Graphite

Pristine graphite (50 mg) was suspended in dry toluene (25 mL) using ultrasonication for 30 min and transferred into 100 mL two necked round bottom flask. Another 100 mL two necked round bottom was charged with NaNO_2_ (6 g) was equipped with pressure equalizer funnel filled with conc. HNO_3_ (6 mL). Further it was connected with graphite containing flask via ground joint connection bent in order to exchange of NO_2_ gas radicals. To the above experimental set up, outlet joint connector was linked to sodium hydroxide (1 N) solution to bubble any excess gas or oxygen. After that conc. HNO_3_ has been added drop by drops into NaNO_2_ containing flask to release NO_2_ radical and it was passed via glass bent to graphite suspension containing flask. Finally, toluene was evaporated and centrifuged using hexane to get purified functionalized [NO_2_]_n_-graphene (45 mg yield).

### 2.4. Synthesis of [OH]_n_-Graphene from [NO_2_]_n_-Graphene

Briefly, NaOH solution (3 N) added drop by drop into 40 mg of [NO_2_]_n_-graphene and sonication for 30 min. After the sonication, the reaction mixture was stirred at 55 °C for 6 h. Further, 20 mL of methanol was added to the reaction mixture at ambient temperature to precipitate the crude black solid product. The crude block solid was washed several times with deionized water to remove any alkaline residues and dried under vacuum at 50 °C for 6 h to obtain hydroxyl functionalized graphene (40 mg yield).

### 2.5. Synthesis of [Br]_n_-Graphene

Briefly, 30 mg of [OH]_n_-graphene was taken in a 100 mL RB flask and 10 mL of dimethyl formamide was added, sonicated for 30 min, to get uniform dispersion. Then, 4.5 mL of triethylamine was added drop by drop to the reaction and allowed to stir for 30 min. The reaction was allowed to cool to 0 °C using ice-salt bath. Further, 4.5 mL of bromoisobutyryl bromide was added very slowly to the reaction mixture and stirred at room temperature for 24 h. The black solid product was centrifuged and washed with water several times and DMF was added in order to neutralize the product. The product was dried under vacuum at 45°C to get [Br]_n_-graphene (25 mg yield).

### 2.6. Synthesis of Graphene-g-PMMA

Briefly, 20 mg of graphene-initiator is dispersed in 1 mL of DMF and 5 mL of deoxygenated MMA was added to the reaction mixture and degassed by four freeze-pump-thaw cycles. Then, 3 mg of copper (5–50 nm) and 5 mL of PMDETA was added after degassed and purged with nitrogen gas and stirred in a thermostatic oil bath at 60 °C. After 24 h, the SET-LRP reaction was terminated by adding 100 mL of hexane. The resulting polymeric precipitate was filtered, redispersed in 50 mL of THF and again precipitated to remove the Cu^II^/PMDETA complex and unreacted monomer. Finally, a pale gray polymeric solid was obtained after vacuum-drying for 30 h (yield 0.35 g).

## 3. Results and Discussion

In order to explore the formation of a well-controlled grafting of PMMA on the surface of graphene, which mediated through SET-LRP, a bromide functionalized graphene initiator was synthesized. In our previous report, we have developed the hydroxyl SWCNTs, from *p*-SWCNT via the addition of nitro radical towards synthesizing the polyurethane nanocomposites via covalent method [30] and the aforementioned methodology was adapted to functionalize the graphene in this present study as well. Further, to facilitate the reaction between -Br and graphene on methyl methacryate monomer, a facile method was introduced for the first time to obtain hydroxyl graphene from graphite via a modified synthetic route [22].

**Figure 1:**
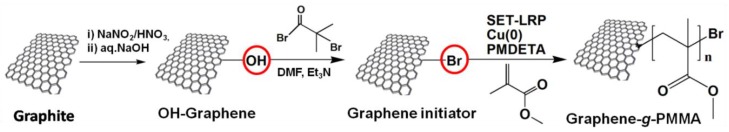
Schematic representation of synthesis of G-*graft* PMMA.

Graphite was rehabilitated into [NO_2_]_n_-graphene by the addition of nitro radical, which was produced from the mixture of sodium nitrite and conc. HNO_3_, followed by alkaline and bromine treatments. This afforded an enhanced yield of [OH]_n_-Graphene and [Br]_n_-Graphene as given in Figure 1. Further, the functionalized [Br]_n_-Graphene was covalently added in trace loadings to deoxygenated MMA monomer in the presence of catalysts such as Cu(0) and *N*,*N*,*N^I^*,*N^II^*,*N^III^*-Pentamethyl diethylene triamine (PMDETA), which essentially to make the graphene to act as radical initiator itself to afford the G-*graft*-PMMA without compromising their unique properties, as shown in Figure 1. As mentioned, a modified methodology was adapted for the synthesis of G-*graft*-PMMA(S) using sacrificial initiator.

### 3.1. FT-IR and UV-Vis Spectroscopy Study

**Figure 2:**
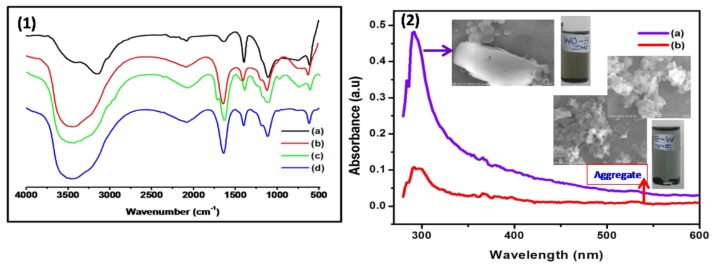
(**1**) FT-IR spectra of (a) OH-graphene, (b) [Br]_n_-Graphene, (c) graphene-*g*-PMMA and (d) graphene-*g*-PMMA(S), and (**2**) UV-*vis* spectra of (a) graphene-*g*-PMMA, (b) graphene-*g*-PMMA(S) with their FE-SEM images.

The FT-IR spectrum of graphene-*g*-PMMA exhibited the characteristic vibration bands for –C–H at 959 cm^−1^–C–O–C at 1109 cm^−1^, –C–H at 1398 cm^−1^, –C=O at 1640 cm^−1^, and –C–OH at 3471 cm^−1^, which confirmed the formation of structure of PMMA on graphene surfaces Figure 2(1). Further, the –C=O stretching vibration of carbonyl is lower shifted to 1623 cm^−1^for graphene-*g*-PMMA(S). A new peak is also appeared at 753 cm^−1^ (–C–H out of plane bending) corresponding to the sacrificial initiator-based polymer nanocomposites and disappeared for graphene-*g*-PMMA. These observations essentially indicated that the polymer end chains are well-polymerized onto the graphene surface in a controlled manner. Also, the bromide functionalization on graphene is confirmed by the presence of alkyl halide group –C–Br at 612 cm^−1^ as depicted in Figure 2(1b).

Further, the functionalization of graphene with PMMA is characterized by UV-*vis* spectroscopy, where the maximum absorption of π–π* Plasmon peak at 289 nm is observed for G-*g*-PMMA(S), whereas the absorption peak is found to be shifted to 300 nm for G-*g*-PMMA. This basically indicated that the conjugative effect of *sp*^2^ cluster in graphene and linking chromophores, such as –C=O and –C–O groups, exist on the graphene surfaces. The greater absorbance and intense peaks were noticed for G-*g*-PMMA, which is attributed to the better hyperchromic effect of the nanocomposites. Further, the aggregation and dispersion states have also been compared with same samples and analyzed by FE-SEM, which showed significant changes in their morphology as shown in Figure 2(2).

### 3.2. Structural Identification of Polymer Nanocomposite

**Figure 3:**
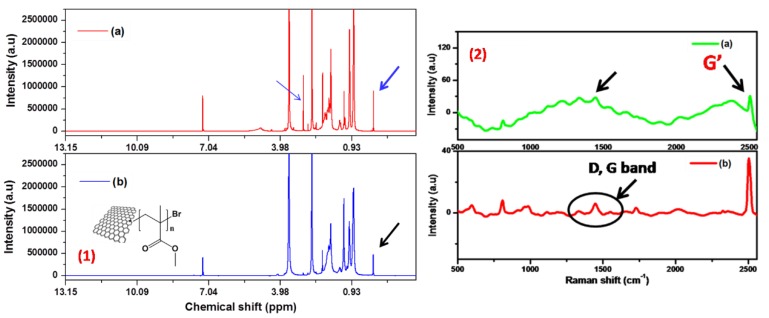
(**1**) ^1^H NMR of (a) graphene-*g*-PMMA(S) and (b) graphene-*g*-PMMA, (**2**) Raman spectra of (a) graphene-*g*-PMMA(S) and (b) graphene-*g*-PMMA.

The ^1^H NMR spectrum of graphene-*g*-PMMA and graphene-*g*-PMMA(S) as given in Figure 3(1) that taken in CDCl_3_, show the peaks at 0.8–1.4, 1.83 and 3.6–4.8 ppm correspond to the protons from the –CH_3_, –CH_2_ and –OCH_3_ groups, which clearly confirmed the growth of PMMA on the graphene surface. Also, the peak around 2.6 ppm corresponds to six protons from (–CH_3_)_2_ confirmed the structure of initiator. Additionally, a new peak at 3.0 ppm (–CH_2_–Br) is noticed for graphene-*g*-PMMA(S), whereas it has disappeared for graphene-*g*-PMMA. Further, the chemical shifts observed in the synthesized G-*g*-PMMA and G-*g*-PMMA(S) are analyzed using the ^13^C NMR to find the monomer units, initiator and chain end group, which usually form during SET-LRP reaction. Accordingly, similar to the alkyl bromide initiator, there are two peaks observed at 19.1 and 29.8 ppm corresponding to methyl groups.

Moreover, concerning the monomer repeat unit, the peak at 42.7 (quaternary carbon) and the resonance at 51.8 ppm (–C–Br) are also observed, where it indicated that the polymerization process is alive as shown in ESI Figure S6. It clearly confirms the polymer grafting on graphene surface. Generally, carbon-based materials such as carbon nanotubes, graphene, and nanodiamond, can be characterized at high Raman intensities, and hence it is a powerful technique to characterize such materials [31]. Raman spectra demonstrated the reliable characteristics concerning the formation of the polymer end chain onto the graphene surface by showing the presence of D (1420 cm^−1^), arising from the stretching of *sp^3^* carbon, G (1547 cm^−1^) arising for *sp*^2^ carbons of graphene sheets, G’ (2501 cm^−1^) and other bands at 1090 cm^−1^ (–C–C), 981 cm^−1^ (–O–CH_3_) and 594 cm^−1^ (–C–C–O), respectively, which confirmed the grafting of polymer on the graphene nanosurfaces (G-*g*-PMMA) (see Figure 3(2)). Also, the disorder band (D band) has been increased from 1410 cm^−1^ to 1420 cm^−1^. The peaks at 594 cm^−1^ (–C–C–O) and 981 cm^−1^ (–O–CH_3_) have disappeared in the case of G-*g*-PMMA(S) sample, which indicated that the polymer has well grown on graphene surfaces.

### 3.3. Matrix-Assisted Laser Desorption Ionization Time-Of-Flight Mass Spectroscopy (MALDI-TOF) Study

MALDI-TOF-MS is one of the reliable and accurate techniques to identify the high molecular weight polymer material and biomolecules. Samira et al., reported that the experimental values of *m/z* of neat PMMA was about 1102.16 *m*/*z*, 2036.91 *m*/*z* and 3569.40 *m*/*z* analyzed by MALDI TOF for PMMA [32]. Justine et al. also reported that the poly (methyl methacrylate) (PMMA) and poly(methyl acrylate) (PMA) based polymeric materials have been synthesized by SET-LRP and ATRP methods using bifunctional initiators (methyl dichloroacetate and dibromoacetate). Further, the chain end functionality of the polymer has been studied in detail by MALDI-TOF mass spectroscopy [33]. Also, MALDI allows the identification of various chain end group, which species present in minor amount in polymer end group identification is so decisive in the polymer analysis. The MALDI-TOF has also granted voluble information about the repeating unit and terminal group of the PMMA, which leads to further clarification of polymerization mechanism and average molecular weight distribution. Moreover, the end groups (–C–Br) of the polymer composite (graphene-g-PMMA) play a significant role in determining their physical and chemical properties. In the initiation step a halogen atom is transferred from the Graphene-initiator to metal catalyst (Cu/PMDETA), which could be oxidized to a higher oxidation state, hence, the initiator undergoes higher conversion.

**Figure 4:**
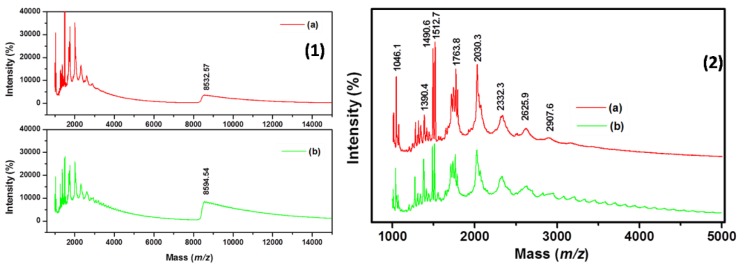
MALDI-TOF of (**1**)–(**2**) (**a**) Graphene-graft-PMMA(S) and (**b**) Graphene-graft-PMMA.

Accordingly, our results are also in support of their results, where the respective peaks G-*graft*-PMMA are observed at *m*/*z* value [1046.1 (–CH_3_), 1390.4, 1490.6, 1512.7, 1763.8, 2030.3 (–CH_3_), 2332.3, 2625.9 and 2907.6 (–CH_3_)]. Additionally, the highest molecular weight is also noticed at 8594.54 *m*/*z*, which indicated that the MMA monomer units are well polymerized onto the graphene surface [see Figure 4 (1)–(2)(a)–(b)].

### 3.4. Surface and Thermal Properties of Polymer Nanocomposites

It is believed that the activation might have occurred by the reaction of alkyl halide (-Br) with Cu(0) and the deactivation might have occurred by the reaction with Cu(II), where itacts as a ‘living-in-nature’ in the polymerization system. This result also supports our previous report, where we have prepared the SWCNTs-*g*-PMMA through radical polymerization with well controlled molecular weight by GPC around *M*_n_ = 8500 with PDI = 1.4 [23]. Accordingly, the performed zeta potential measurements in this study also showed a well-dispersion behaviour as well as improved charge stability as the [Br]_n_-graphene is employed as the initiator for the controlled polymerization, where it was also compared with sacrificial initiator as well. There is a dramatic increase in the positive zeta potential (ζ) value from 1.38 to 1.74 mV for the G-*g*-PMMA(S) and G-*g*-PMMA, respectively. The polydispersity index (PDI) and particle size values have been found to be gradually decreased for G-*g*-PMMA (PDI:0.381, 246 nm) and increased for G-*g*-PMMA(S) (PDI:0.784, 474 nm).

**Figure 5:**
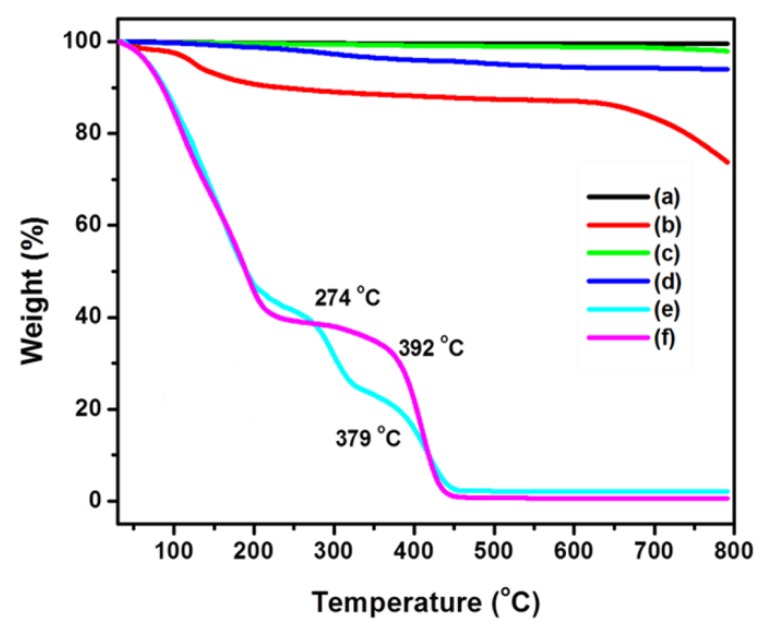
Thermogravimetry analysis (TGA) of (a) GO, (b) [Br]_n_-Graphene, (c) [NO_2_]_n_-Graphene, (d) [OH]_n_-Graphene, (e) graphene-*g*-PMMA(S) and (f) graphene-*g*-PMMA.

It should be noted that the observed higher polydispersityvalue implies that there is some uncontrolled end chain happened during the polymerization reaction, which may be mediated through the sacrificial initiator. On the other hand, the G-*g*-PMMA sample has a uniform growth of polymer on the surface with better dispersion and possesses monodispersed behaviour as shown in ESI Figures S1–S4. This Cu(0) mediated radical polymerization method has received great interest due to their controlled polymerization rate at low temperature. On the other hand, the thermogravimetric analysis further provided a supportive evidence for the complete covalent grafting of PMMA on graphene. The thermogravimetric analysis (TGA) of graphite and [NO_2_]_n_-Graphene is found to be stable up to 790 °C with weight loss around 6.52% and there is no decomposition due to strong π-π interaction. However, the [OH]_n_-graphene started to decompose slightly at 250 °C due to the presence of oxygen-containing groups that usually decomposed between 250–300 °C, whereas, the [Br]_n_-Graphene decomposition started at 150 °C and remained stable up to 677 °C with weight loss of 24.7%. Such variation is essentially occurred due to the presence of alkyl bromide moiety on graphene. The thermal stability of graphene-*g*-PMMA(S) and graphene-*g*-PMMA can be well-compared. From the results, two stages of degradation can be observed for graphene-*g*-PMMA(S). Accordingly, the first stage onset decomposition at 224 °C (43.65% residue) and second degradation at 348 °C (23.28% residue), which may have occurred due to the removal of polymeric unit, which is found to be stable up to 435 °C. In the case of graphene-*g*-PMMA, it has two stages of decompositions and stable up to 445 °C with weight loss residue of 1.9% as shown in Figure 5. The TGA trace for G-*g*-PMMA shows a considerable shift of the weight loss towards higher temperature with a thermal stabilization up to 10 °C, which is higher than that of the G-*g*-PMMA(S) that may be due to the controlled and covalent addition of graphene. Apparently, considering the amount of oxygen-containing hydroxyl and bromide groups, the amount of *sp^3^* hybridized carbon decreased with increasing amounts of *sp^2^* hybridized carbon, which suggested that the conjugated graphene structure has been fully grafted with polymer chain through SET-LRP [34,35,36,37,38,39]. Further, the glass transition and melting temperatures is analyzed by DSC for G-*g*-PMMA [*T*_g_: 88 °C, *T*_m1_: 102 °C and *T*_m2_: 378 °C] and G-*g*-PMMA(S) [*T*_g_: 75 °C, *T*_m1_: 95 °C and *T*_m2_: 287 °C], which revealed that the glass transition is lowered up to 10 °C. Further, the two-stage melting behaviour is also observed, where the first and second melting temperature is increased from 13 °C to 91 °C for G-*g*-PMMA (see Supplementary Figures S7 and S8). In addition, the thermal properties (*T*_g_ and *T*_m1_ and *T*_m2_) have also been enhanced upon the covalent addition of [Br]_n_-graphene into the polymer matrix, which may be due to a restriction in the main chain mobility and the confinement effect of nanofillers (graphene).

### 3.5. Surface Morphological and Hydrophobic Properties of Polymer Nanocomposite

**Figure 6:**
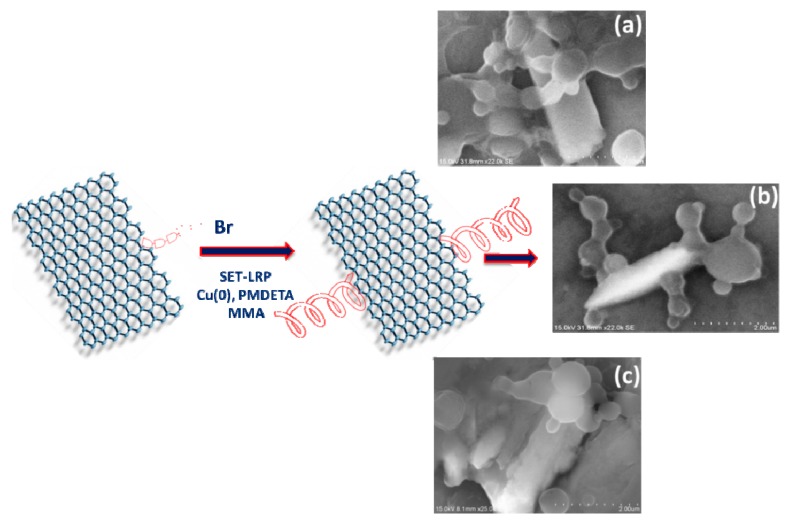
FE-SEM images of (**a**–**c**) graphene-*g*-PMMA.

Generally, GO possesses the hydrophilic functional groups such as –OH, –COOH and epoxide, which can promote the intercalation of water molecules into graphene gallery, thus produce highly dispersible GO in aqueous medium. In this reaction, the alkyl initiating groups are introduced onto the graphene surfaces by esterification reaction between hydroxyl groups and bromoisobutyryl bromide, where it further reacted with methyl methacrylate monomer via SET-LRP and produced highly dispersed GO, where it is found to be soluble in polar and non-polar solvents with a colour change from brown to black during the radical polymerization. This essentially revealed that some structural changes occurred in the graphene surfaces. Hence, the polymer end chain is rapidly grown on graphene surface with excellent dispersion, where it appeared like the polymer macromolecules are wrapping on their surfaces (see Figure 6a–c). In addition to this, the aggregations have also been occurred in G-*g*-PMMA(S) material due to uncontrolled polymerization on the graphene surface, which is in good agreement with the FE-SEM and photographic images as shown in Supplementary Figure S5a,b.

**Figure 7:**
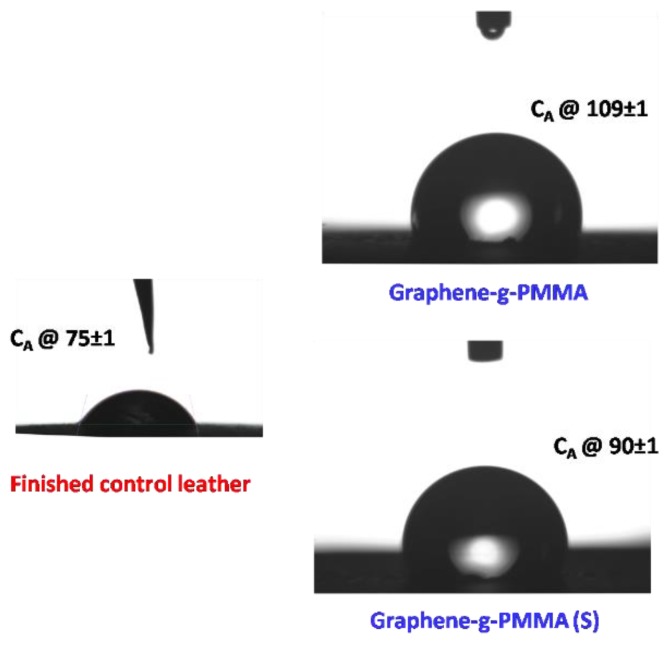
Water contact angle measurement of finished control leather, graphene-*g*-PMMA and graphene-*g*-PMMA (S).

In this context, we have also developed the graphene-*g*-PMMA with the enhanced dewettingbehaviour (contact angle (C_A_) @109±1 (30 min) with respect to the water droplet on the leather surface showing better hydrophobic properties. Notably, materials with such characteristics can be pertinent to different applications, including the smart hydrophobic leather accessories. Further, water contact angle on the ‘finished’ control leather has been observed to be CA@75±1 (30 min) and graphene-*g*-PMMA(S) CA@90±1 (30 min). From these results, lower wetting behaviour is realized for graphene-*g*-PMMA(S) as compared to the graphene-*g*-PMMA (see Figure 7). This essentially implied that the graphene-initiator is well polymerized and their optical, morphological, and thermal propertiesenhanced. The obtained results have also demonstrated that the underlying graphene substrates do not affect the wettability [27,28,29].

## 4. Conclusions

In conclusion, the effect of the sacrificial initiator and graphene based radical initiator was successfully surface polymerized using a controlled SET-LRP. The presence of bromoisobutyryl bromide based sacrificial initiator that generally produced the uncontrolled polymerization was evident by the cross-linking behavior as observed in FE-SEM images, and it also clearly confirmed the negative effect of sacrificial initiator during polymerization. Further, the presence of polymer backbone chains onto the graphene surface through complete grafting was confirmed via FT-IR, UV-vis spectroscopy, NMR, Raman, MALDI-TOF, DSC and TGA techniques. Furthermore, the functionalization of graphene through polymer was found to endow the better dispersion and improved zeta potentials, as evident by the dispersion measurements. The synthesized polymeric materials were coated on the leather surfaces and showed enhanced hydrophobic properties. Thereby, this novel nanocomposite material synthesized in the present work can be used for various damping and coating applications.

## Supplementary Materials

The following are available online at https://www.mdpi.com/2073-4360/12/4/874/s1.
