# Supplementary material for: Copper (0) Mediated Single Electron Transfer-Living Radical Polymerization of Methyl Methacrylate: Functionalized Graphene as a Convenient Tool for Radical Initiator
Source: Polymers (Basel). 2020 Apr 10;12(4):874. doi: 10.3390/polym12040874 (PMC7240427; doi:10.3390/polym12040874)
Supplement: Supplementary file 1 [file polymers-12-00874-s001.pdf]

polymers

MDPI

Supplementary Materials

Copper (0) Mediated Single Electron Transfer-Living Radical Polymerization of Methyl Methacrylate: Functionalized Graphene as a Convenient Tool for Radical Initiator

Adhigan Murali [1,*], Srinivasan Sampath [2], Boopathi Appukutti Achuthan [3], M. Sakar [4,*], Suryanarayanan Chandrasekaran [5], N. Suthanthira Vanitha [6], R. Joseph Bensingh [1], M. Abdul Kader [1], Sellamuthu N. Jaisankar [3]

[1] School for Advanced Research in Polymers (SARP)-Advanced Research School for Technology and Product Simulation (ARSTPS), Central Institute of Plastics Engineering & Technology (CIPET), Ministry of Chemicals & Fertilizers, Govt. of India, Chennai 600032, India; josephbensingh@gmail.com (J.B.); kader36@yahoo.com (M.A.K.)
[2] Department of Materials Science, School of Technology, Central University of Tamil Nadu, Thiruvarur 610101, India; sampathsrinivasan@yahoo.com
[3] Polymer Science and Technology Division, Council of Scientific and Industrial Research (CSIR)-Central Leather Research Institute (CLRI), Adyar, Chennai 600020, India; aaboopathichem@gmail.com (B.A.A.); snjaio@yahoo.com (S.N.J.)
[4] Centre for Nano and Material Sciences, Jain University, Bangalore 562112, Karnataka, India.
[5] Faculty of Pharmacy and Pharmaceutical Sciences, University of Alberta, Edmonton, AB T6G 2E1, Canada; jobforsurya@gmail.com
[6] Department of Electrical & Electronics Engineering, Muthayammal Engineering College (Autonomous), Namakkal 637408, Tamilnadu, India; varmans03@gmail.com
* Correspondence: precymurali@gmail.com (A.M.); m.sakar@jainuniversity.ac.in (M.S.); Tel.: (+91)44 22254794 (A.M.); Fax: (+91)44 22254793 (A.M.)

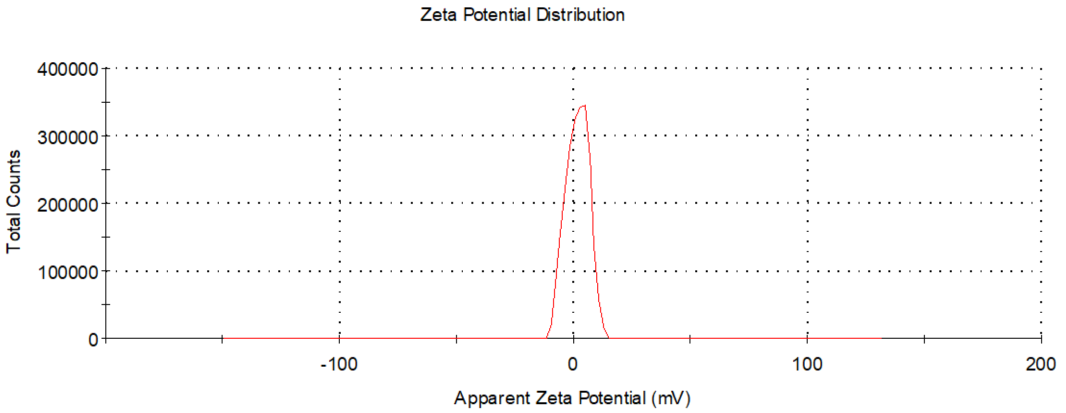

Figure S1. Zeta potential of Graphene-graft-PMMA (PDI:0.381, 246 nm).

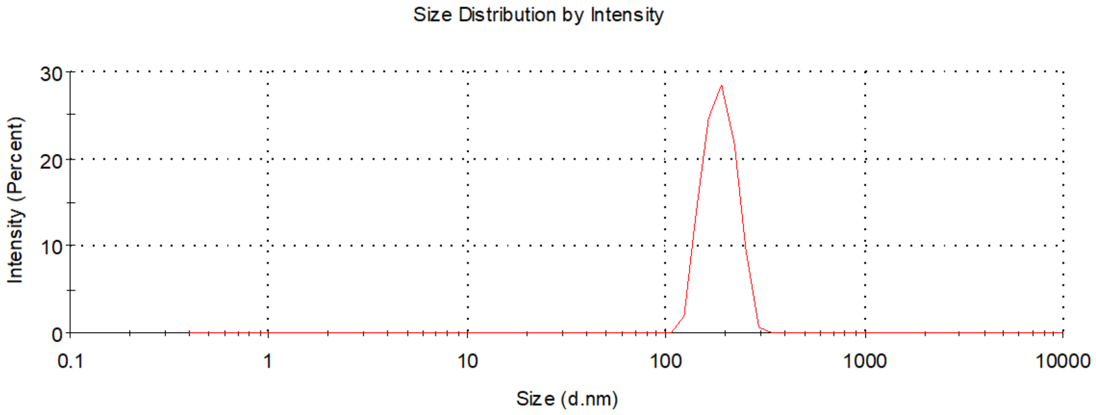

Figure S2. Particle size distribution of Graphene-graft-PMMA (PDI:0.381, 246 nm).

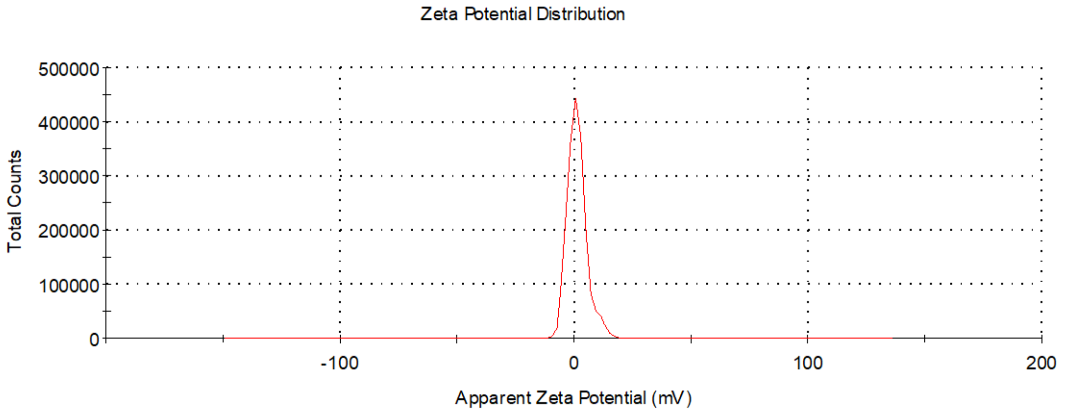

Figure S3. Zeta potential of Graphene-graft-PMMA(S) (PDI:0.784, 474 nm).

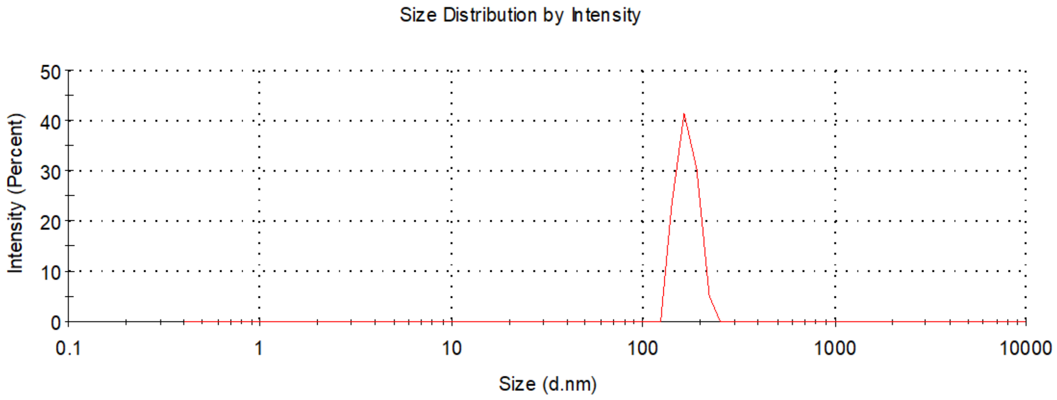

Figure S4. Particle size distribution of Graphene-graft-PMMA(S) (PDI:0.784, 474 nm).

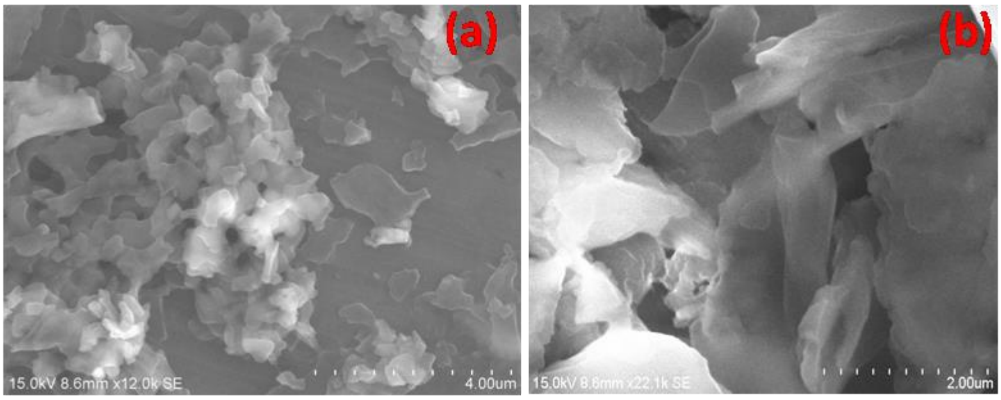

Figure S5. FE-SEM images of G-g-PMMA(S).

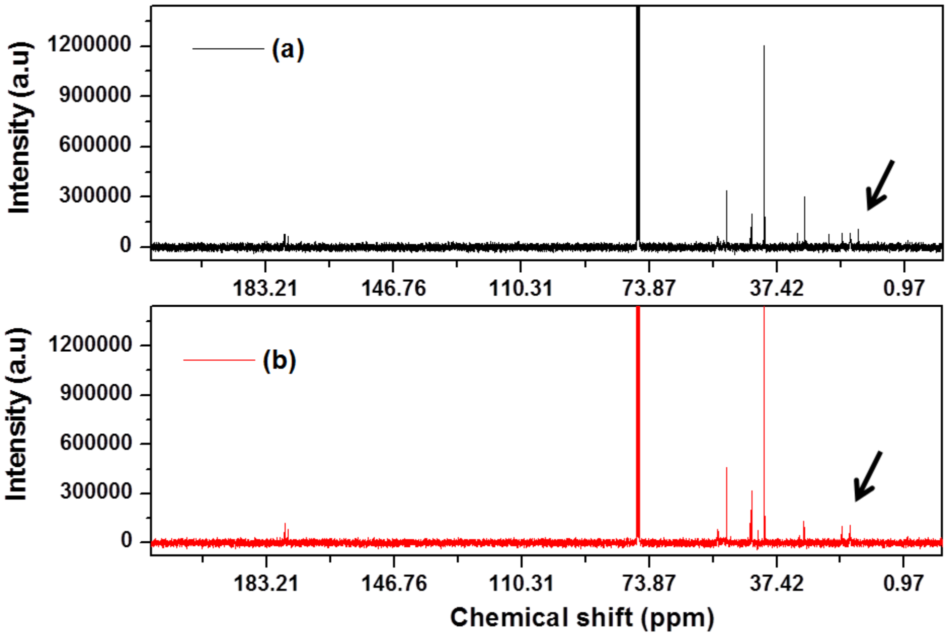

Figure S6. ^{13}C NMR of (a) graphene-*g*-PMMA(S) and (b) graphene-*g*-PMMA.

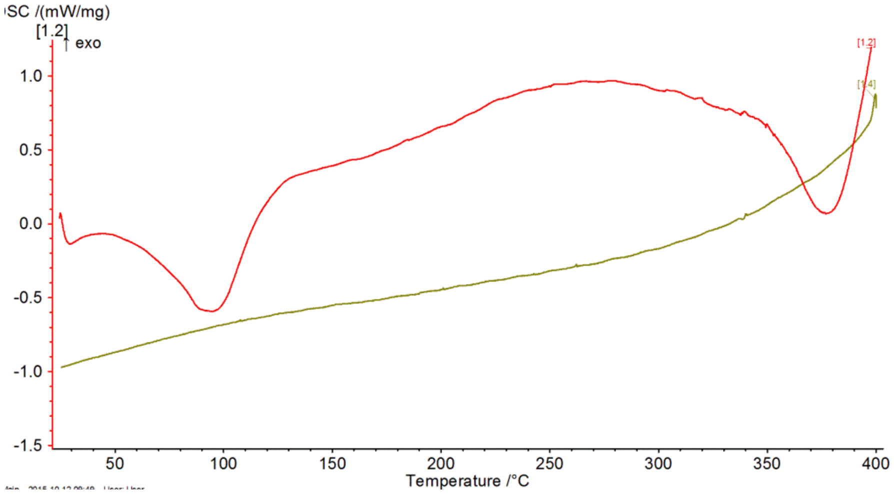

Figure S7. DSC data for Graphene-graft-PMMA.

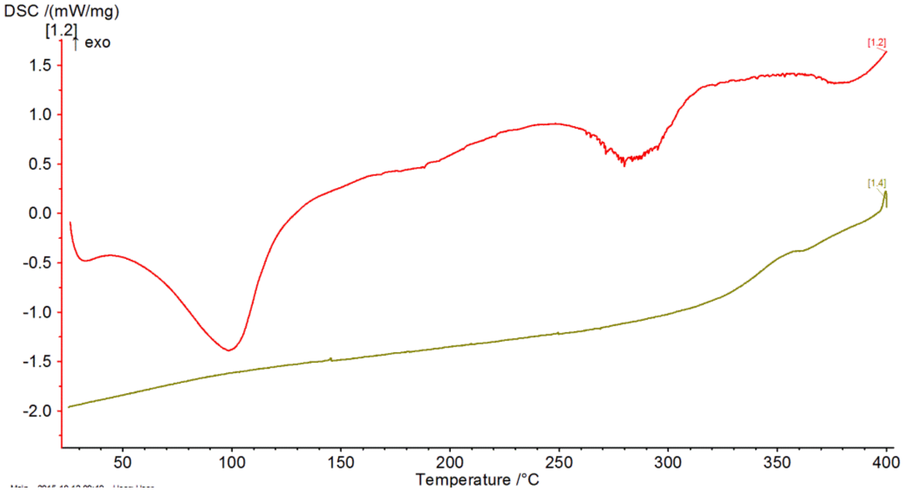

Figure S8. DSC data for Graphene-graft-PMMA(S).
